# Investigating the effect of a 3-month workplace-based pedometer-driven walking programme on health-related quality of life in meat processing workers: a feasibility study within a randomized controlled trial

**DOI:** 10.1186/s12889-015-1736-z

**Published:** 2015-04-22

**Authors:** Suliman Mansi, Stephan Milosavljevic, Steve Tumilty, Paul Hendrick, Chris Higgs, David G Baxter

**Affiliations:** School of Physiotherapy, University of Otago, Dunedin, New Zealand; School of Physiotherapy, University of Saskatchewan, 1121 College Drive, Saskatoon, SK S7N 0W3 Canada; Division of Physiotherapy Education, The University of Nottingham, Nottingham, NG5 UK

**Keywords:** Physical activity, Pedometers, Walking intervention, Quality of life, Step count, Workplace, Ambulatory activity

## Abstract

**Background:**

In New Zealand, meat processing populations face many health problems as a result of the nature of work in meat processing industries. The primary aim of this study was to examine the feasibility of using a pedometer-based intervention to increase physical activity and improve health-related outcomes in a population of meat processing workers.

**Methods:**

A single-blinded randomized controlled trial (RCT) was conducted. A convenience sample of meat workers (n = 58; mean age 41.0 years; range: 18-65) participated in the trial. Participants were randomly allocated into two groups. Intervention participants (n = 29) utilized a pedometer to self monitor their activity, whilst undertaking a brief intervention, and educational material. Control participants (n = 29) received educational material only. The primary outcomes of ambulatory activity, and health-related quality of life, were evaluated at baseline, immediately following the 12-week intervention and three months post-intervention.

**Results:**

Fifty three participants completed the program (91.3% adherence). Adherence with the intervention group was high, 93% (n = 27/29), and this group increased their mean daily step count from 5993 to 9792 steps per day, while the control group steps changed from 5788 to 6551 steps per day from baseline. This increase in step counts remained significant within the intervention group p < 0.005; at three months post-intervention representing a 59% increase over baseline scores. There were significant group changes with large effect sizes for step count change (d = 1.94) and self-reported physical activity (p < 0.005; d = 2.59) at 12 weeks intervention. Further, results showed non-significant between-group differences in physical component (PCS) and mental component (MCS) scores (PCS: p = 0.44; MGD = 0.99, 95% CI, -1.6 to 3.6; ES = 0.14, and MCS: p = 0.90, MGD = 0.15; 95% CI, -2.3 to 2.6, ES = 0.022) at 12 weeks intervention.

**Conclusions:**

This research provides important information for a larger (RCT) in the future: results demonstrated that a pedometer-driven walking intervention in combination with goal setting, and self-monitoring supported by weekly e-mails are feasible and potentially effective in increasing step count within the workplace setting over the short term.

**Trial registration number:**

Australian New Zealand Clinical Trials Registry (ANZCTR) ACTRN12613000087752.

## Background

Daily physical activity is defined as “*any bodily movement produced by the skeletal muscle that results in energy expenditure”* [1]. Moderate physical activity from walking is considered beneficial in the prevention and management of various chronic diseases including: obesity, high blood pressure, diabetes mellitus, musculoskeletal disorders (MSD), and cardiovascular disease [2-4], and is associated with a reduction in premature mortality and improvement in quality of life [5]. Despite such evidence, more than 31% of adults do not take part in recommended levels of physical activity [6,7] leading to potential increases in health risks related to insufficient physical activity, and a likely increased economic burden on the health care system. Increasing levels of physical activity to help inactive individuals become more active, has considerable potential for reducing the burden of chronic diseases and improving health- related quality of life (HRQL).

In developed countries, modern conveniences and technology have contributed to increasing physical inactivity among adults. For example, World Health Organization (WHO) reported in 2008, 31% of adults exhibit a sedentary lifestyle, and have a 20-30% increased risk of mortality compared to active people [6,7]. Physical inactivity in New Zealand (NZ) is a significant public health issue [6] with 46 per cent of adults not meeting current recommendations for daily physical activity [8]. Worldwide, the highest rates of inactivity are among workplace adults [9,10], with 79% of US employees worked at sedentary- and light-intensity jobs; represented approximately11 hour per day in sedentary behaviors [11]. For NZ adult workers, previous research [12] has reported a high prevalence of inactivity (57%) measured by pedometer across six different workplace settings. Meat-processing workers in NZ are an ageing population [13] with consequential health-related issues consistent with an ageing workforce, a sedentary lifestyle, and chronic disease that include obesity, musculoskeletal disorders, hypertension, diabetes, and other cardiopulmonary problems [13-15].

In developed countries, most workers spend about a third of their waking hours in the workplace; therefore, the workplace is recognized as an ideal setting for health promotion and physical activity strategies [16-18]. Through the workplace there is potential to improve health status by increasing the level and capacity for a more physically active lifestyle, which may link to a reduction in occupational injuries and protection of workers from accidents, reducing working hours lost as a result of absence due to illness or injury, as well as reducing the costs of treatment and claims for compensation [19]. A number of systematic reviews in a variety of workplace settings support the effectiveness of physical activity interventions for improving overall health [20-23]. A recent systematic review investigated 58 studies using mixed strategy interventions such as counselling/support, promotional messages/information and physical activity/exercise interventions to promote physical activity in the workplace [18]. The results show some evidence that workplace physical activity interventions can be efficacious in promoting physical activity when compared to control groups receiving no intervention.

Pedometer based waking interventions have been widely used to increase the level of physical activity and improve health-related outcomes in the general population [24,25] and in the workforce [21,23]. The use of social cognitive methods incorporating self-efficacy, goal setting, feedback [25-27] and behavioural support materials about the health benefits of physical activity interventions [28,29] are also considered effective strategies to increase physical activity in these populations [24,26,30,31]. A recent systematic review of interventions delivered in workplace settings reported that pedometer interventions incorporating activities at social and environmental levels were more likely to report successful outcomes than those that did not have these components [23].

To our knowledge, no study has employed pedometer-driven walking as a motivational strategy and intervention together with goal setting in order to increase daily ambulatory activity among meat processing workers. The primary aims of this study were to examine the feasibility of using a pedometer-based walking intervention, incorporating a brief intervention, along with educational material and email support to increase ambulatory activity and improve health-related outcomes in a population of meat processing workers when compared to a control group receiving educational material alone. We hypothesized that the pedometer-driven walking intervention would be a feasible tool to increase participants’ daily ambulatory activity levels and improve health outcomes compared to a control group.

## Methods

### Study design and ethics

This was a feasibility study using a randomized controlled trial design, which collected data at three time points (baseline, 12 weeks (at conclusion of intervention), and three months post-intervention). The study was approved by The Otago Human Ethics Committee, School of Physiotherapy (Protocol number12/313) and written informed consent was obtained from all participants prior to beginning the study. The study protocol has been published elsewhere [32] and was registered in the Australian New Zealand Clinical Trials Registry (ANZCTR) under reference no:ACTRN12613000087752.

### Participants and setting

Adults over the age of 18 years were recruited from a large meat processing plant in the South Island of New Zealand. Employee recruitment was through advertisements (posters) in different work-sites including the on-site health clinic, plant administration, cafeterias, and all department notice-boards. Potential participants (n = 95) were screened for eligibility for entry into the randomized control trial by wearing the pedometer (Yamax Digi-walker SW-200) for seven consecutive days and using the physical activity readiness questionnaire (PAR-Q). Inclusion criteria were: not regularly physically active (less than 7,500 steps per day) [30]; able to walk continuously for at least 10 minutes; able to read and sign an informed consent form and questionnaires and willing to participate for the full study duration.

### Randomization

**Figure 1:**
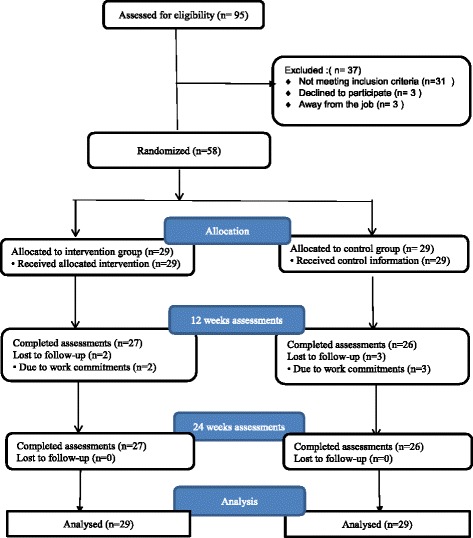
CONSORT diagram of participant flow through the programme.

Sixty participants were eligible to participate in the study; however two participants dropped-out leaving 58 participants who were randomized into the two groups (i) pedometer-driven walking (PW; n =29), (ii) control group receiving educational material alone (CG; n = 29). A sample randomization was performed using sealed envelopes by an independent person not linked to the study. Participants were invited to choose an envelope from a basket containing envelopes that allocated 50% of the sample to the intervention and 50% to the control group. Each envelope contained the group name for allocation, and timetable of the study. Researchers and participants were not blinded to group allocation. A registered nurse, blinded to group allocation, performed assessment at baseline, 12 weeks (at the conclusion of the intervention), and three months post-intervention. The flow of participants through the recruitment process and randomization is presented in (Figure 1) according to the recommendations of the CONSORT statement [33].

### Preparation of participants

At baseline, participants were instructed to wear the pedometer (Yamax Digi-walker SW-200) on the waistband of their clothing for seven days, based on previous study protocols [34-38] in order to establish baseline step-counts during normal daily activity. Participants were instructed on how to wear and use the pedometer during all waking hours, except for periods immersed in water (bathing, swimming), during certain sporting activities (playing basketball or soccer, etc.), or in bed at night. They were instructed to reset the pedometer to zero at the beginning of each day, and remove it at the end of each day, record on a step calendar the date and the time the pedometer was attached and also removed, and the total number of steps displayed on the pedometer at the end of each day.

After randomization, all participants attended a 30 minute education session on the health benefits of being physically active, after which participants in the walking group additionally received a brief intervention group session of up to 70 minutes, including a 10 minute self-efficacy walk, a 30 minute session focussing on physical activity behaviour change, and 30 minutes focussed on the education resource material (physical activity booklet). This intervention session was based on the Back 2 Activity protocols [39] and conducted by physiotherapists (including one with training in motivational interviewing), and the researcher.

### Intervention protocol

The 12 week pedometer-driven walking intervention was based on self-regulation theory (SRT) [40] and included goal setting, feedback, educational material, and the use of a step calendar for self-monitoring. Participants were required to walk for at least five days per week to meet evidence based international guidelines that recommend adults to accumulate at least 30 minutes of moderate intensity activity, on at least five days/week, to achieve optimal health benefits [41]. The intervention was performed during working hours and/or leisure time on the week.

#### Educational materials

Participants in both intervention and control groups received standardized educational materials (physical activity justification booklet of “Walk into Health” Toronto Public Health www.toronto.ca/health/walkintohealth) which consisted of written and graphical information describing the importance of walking as a physical activity for health benefits and prevention of disease [2,3].

#### Goal setting

At the beginning of each week, participants received a weekly email reminder about his or her step-count goal for that week, based on their baseline walking activity level. The aim was to gradually increase the level of activities by 5% from their previous goal setting target, with an overall goal to reach at least 10,000 steps per day at the end of the 12 week period. These targets are based on international guidelines for walking interventions [42]. However, those who reached 10,000 steps per day (at any time during the program) were also encouraged to maintain and increase their physically active lifestyle.

#### Step count and feedback

Participants in the intervention group received permanent step-count feedback through the digital display on the pedometer. Participants also received personalized weekly emails about daily average step-counts and additional health information, to encourage their adherence with the program.

#### Step calendar

Participants in the intervention group were given a diary to record their step count and note their adherence to the programme, the time of day, duration of the walk, the week’s step-count goal, and the number of steps taken at the end of each day.

#### Control group

Participants randomly allocated to the control group were encouraged to read the educational activity material. At the completion of the 12 weeks, these participants again wore the pedometer for one week to establish a weekly step count for comparison to baseline scores.

### Outcome measurements and methods

Outcome measurements were made at baseline, immediately after the 12 week pedometer-driven walking programme, and at the three months post-intervention time points. The primary outcome measurement were feasibility outcomes, physical activity level, health-related quality of life, and physical fitness. Secondary outcome measures were blood pressure (BP), body mass index (BMI), body fat percentage (BF), self-efficacy, and waist circumference (WC).

#### The feasibility and acceptability of using pedometers

Participants’ adherence to the pedometer-driven walking programme was evaluated by analysing the pedometer logs to determine the number of days that the pedometer was worn and dividing by the total number of intervention days, and also the number of hours of use per day over the 12-week period. Participant satisfaction with the intervention was evaluated by using survey questions to explore opinions regarding intervention components. The questions included participation in the intervention, satisfaction with participation, and pedometer usage after completing the intervention. In addition, participants were asked to report any adverse events as well as provide any comments about the intervention procedure in a series of open-ended questions.

#### Physical activity levels (PA)

Objective change in PA level was measured using the Yamax Digi-walker SW-200 (Yamax, Tokyo, Japan) pedometer. This model demonstrates acceptable reliability for research purposes in the adult population [43,44], found to be an accurate pedometer in counting steps, recording between 1-3% error within both free living and controlled laboratory settings [45-47]. In addition, participants also completed the International Physical Activity Questionnaire Short Form (IPAQ-SF) self-report, to report the frequency (days per week), duration (minutes), and level of intensity PA across a variety of different domains. The questionnaire consists of seven items which provide information about activity at various intensity levels during the previous week including aerobic activities (vigorous intensity), cycling activities (moderate intensity), walking activities, and sitting time undertaken [48,49]. It was developed as an instrument to measure health-related physical activity in working age populations, and is considered a valid and reliable measure for monitoring population levels of physical activity [48,50,51].

#### Health-related quality of life

All participants completed The Short Form 36 version 2 (SF-36v_2_) questionnaire at three time points during the study period. The SF-36v_2_ has been widely used to measure quality of life in general and specific populations [52,53]. It includes 36 questions and eight sub-scale domains of health-related quality of life, which are grouped into two components: a physical component score (PCS) and a mental component score (MCS). The scores on all sub-scales range from 0 (worst score) to 100 (best score) (T-score transformation with mean, 50 ± 10 SD) [54]. The SF-36 questionnaire has been widely studied and reported to be a valid and reliable measure [55] of physical and mental health with good utility that can be completed in five to ten minutes [53,56].

#### Functional exercise capacity

The Six Minute Walk Test (6MWT) was conducted according to the guidelines from the American Thoracic Society. The 6MWT is a self-paced task that has been used to assess functional exercise capacity within a variety of chronic conditions, as well as in healthy adults [57,58]. It is a practical and simple test which does not require expensive equipment or advanced training for technicians, and only requires a 30 metre walkway. It has been shown to have good reliability and validity when used to assess functional capacity [59,60].

#### Anthropometric and physiological measures

During baseline, 12-week, and three months post-intervention assessments, several secondary measures were obtained in the intervention and control groups. Body fat percentage was formulaically measured using skinfold thickness (the Harpenden Skinfold Caliper W/Software) which was taken from four sites (triceps, biceps, subscapular, and suprailiac) according to recommended locations and technique [61-63]. Weight (kg) was measured by digital scales (Terraillon Lovely Classic Electronic Bath Scale) to the nearest 0.1 kg wearing light clothing without shoes on a hard flat surface for accurate measurement. A stadiometer was used to measure height (Seca 213 Portable Stadiometer) without shoes to the nearest 0.1 cm. Blood pressure was measured with an Omron MX3 plus Blood Pressure Monitor (HEM-7200-E) [64] on three occasions with a rest period of one minute between measurements, then an average was taken and recorded, based on previous study protocol [65]. Waist circumference was measured using plastic tape by placing it around the waist at the level of the umbilicus (iliac crest) while participants were standing balanced on both feet, with both arms hanging freely. Three measurements to the nearest 1.0 mm were taken, averaged then recorded on the report survey. Each participant’s height and weight were used to calculate body mass index (BMI) using the following formula: mass (kg) / (height (m) 2).

### Statistical analysis

Data were presented as means, confidence intervals and standard deviations for continuous variables. Independent t-tests were used for nominal data to test for significant differences between the experimental and control groups at baseline. Data were analysed using Microsoft Excel® and Statistical Package for Social Sciences (SPSS®) version 21.0, and two tailed p values of <0.05 were considered statistically significant. A repeated measure, mixed model, ANOVA was used to examine pre- and post- between group differences in all outcomes at each follow-up time point of the study. Bonferroni confidence interval 95% (CI) was used on estimated marginal means at each follow-up time point to show the range of variation for between-group interactions. Within-group changes were assessed using pairwise comparisons for each variable. Effect sizes were calculated using standardised effect sizes for Cohen d values: 0.2 for small effect, 0.5 for medium effect, and 0.8 for large effect [66].

## Results

Of the 58 participants who were randomly allocated to either the intervention or the control group. Fifty three participants completed all of the assessment follow up points. Five participants dropped out after randomization due to work commitments: three from the control group completed only the baseline assessment, while two participants dropped out from the intervention group. In the intervention group, one participant provided pedometer data for three weeks, and the other completed six weeks of pedometer data An intention to treat protocol was performed by replacing missing values with the group mean at 12 and 24-week follow-up time points, giving a final analysis of 29 participants in each group [67] (Figure 1, flow chart). Descriptive characteristics at baseline are shown in Table 1 with no comparative significant differences in descriptive characteristics identified at baseline.

**Table 1:** **Baseline characteristics of participants**

**Characteristic**	**Randomized (n = 58)**		
	**Intervention (n = 29)**	**Control (n = 29)**	**P value**
Age (yr), mean **(SD)**	43 (14.9)	40 (12.2)	0.731
Height (cm), mean **(SD)**	164.4 (11.2)	165 (10.2)	0.512
Gender, n males **(%)**	10 (34.5)	14 (48.3)	0.451
Weight	80.2 (16.9)	76.9 (13.9)	0.418
Step-count, mean (SD)	5993 (1234)	5788 (1172)	0.519
SBP	125.1 (16.3)	122.4 (11.3)	0.474
DBP	76.3 (9.6)	75.0 (6.9)	0.568
Body fat	29.6 (6.9)	27.7 (6.9)	0.306
BMI	29.9 (7.2)	28.3 (4.4)	0.299
WC	98.9 (12.7)	93.5 (12.1)	0.105
6MWT	555 (71.9)	554 (69)	0.969
W.MET	182 (140)	168 (118)	0.694
T.MET	566 (184)	530 (250)	0.532
PCS	49.2 (7.2)	50.5 (8.0)	0.531
MCS	50.3 (8.0)	51.0 (5.7)	0.716

WMET = walking metabolic equivalent, Total MET = total metabolic equivalent, PCS = physical component score, MCS = mental component score, 6MWT = 6 minute walk test, DBP = diastolic blood pressure, SBP = systolic blood pressure, WC=waist circumference, SD = standard deviation.

### Feasibility of using pedometers

Fifty-three of 58 participants completed the programme and satisfaction survey questions, giving an overall adherence rate of 91%. Adherence with the intervention and control groups was high, 93% (n = 27/29) and 90% (26/29) respectively. Participants used the pedometer for a mean of 6.7 (±0.2) days out of 7 over the 12-week study period; the mean number of hours of use per day was 13.8 ± 0.5 hours. Satisfaction scores with the intervention were high overall, with a median score of 4 or 5 out of 5 on a 5-point likert satisfaction scale for all questions. Overall, the majority of participants reported that the pedometer was easy to use, while 17/27 participants reported that supporting materials helped them increase their daily physical activity. The majority of participants (16/27) indicated that they would continue to use the pedometer to increase their activity in the future, and all participants reported using the pedometer for 10 weeks or greater with no serious adverse effects reported.

### Physical activity and fitness

Repeated measures ANOVA analysis showed a statistically significant time by group interaction identified in daily step count over time (p < 0.005; F = 142.80). Within group pairwise comparisons revealed the step-count increased from a mean of 5993 (±1234) steps per day during week 0 (baseline) to 9792 (±2053) steps per day by week 12 intervention p < 0.005, or an absolute increase of mean difference MD = 3799 steps (95% CI, 3225 to 4371) in the intervention group. This increase in step counts remained significant within the intervention group (p < 0.005; MD = 3651, 95% CI, 2950 to 4354) at three months post-intervention representing a 59% increase over baseline scores. The control group also showed a significant increase in daily step-count (p = 0.013) from baseline to 12 weeks intervention (MD = 763; 95% CI, 137 to 1388) steps per day (Table 2). A univariate analysis of variance revealed significant between-groups differences in step-count (p < 0.005, effect size ES = 1.94). Data in both intervention and control groups for self-reported physical activity (IPAQ-SF) were converted into metabolic equivalent minutes per week (METs). Repeated measures ANOVA revealed a significant interaction for walking metabolic equivalent (W.MET): p < 0.005; F = 88.26. There was a significant increase in the W.MET with the pedometer group (p < 0.005) compared to the control group (p = 0.545) at 12 weeks intervention .Results shows that there were a significant differences between the groups in the W.MET after the intervention (p = 0.001; ES: 2.57).

**Table 2:** **Primary outcome measures: Mean (SD) of groups changes, and mean differences within group for outcomes between baseline and follow-up periods**

**12 weeks changes**	**Intervention n = 29**				**Control n = 29**			
	**Baseline**	**12 weeks**	**Means difference (95% CI)**	**P value**	**Baseline**	**12 weeks**	**Means difference (95% CI)**	**P value**
Step-count	5993 (1234)	9792 (2053)	3799 (3225 to 4371)	0.005	5788 (1172)	6551(1154)	763 (137 to 1388)	0.013
W.MET	182 (140)	1035 (444)	853 (659 to 1047)	0.005	168 (118)	188 (135)	20 (-63 to 103)	0.545
Total MET	566 (184)	1469 (524)	903 (683 to 1124)	0.005	530 (250)	538 (254)	8 ( -146 to162)	0.898
PCS (0-100)	49.3 (7.2)	53.3 (5.3)	4.0 (0. 9 to 7.1)	0.008	50.5 (8.1)	50.0 (7.1)	−0.5 (-3.5 to 2.5)	0.670
MCS (0-100)	50.3 (8.0)	52.7 (5.2)	2.4 (-0.1 to 5.7)	0.082	51.0 (5.8)	51.7 (7.1)	0.7 (-2.0 to 3.5)	0.519
6MWT	555 (72)	587 (69)	32.6 (20.3 to 44.9)	0.005	554 (69)	569 (74)	14.9 (-6.0 to 36.0)	0.081
**24 weeks changes**	**Intervention n = 29**				**Control n = 29**			
	**Baseline**	**24 weeks**	**Means difference (95% CI)**	**P value**	**Baseline**	**24 weeks**	**Means difference (95% CI)**	**P value**
Step-count	5993(1234)	9645(1906)	3652 (2950 to 4354)	0.005	5788 (1172)	6266 (1648)	478 (-306 to1263)	0.396
W.MET	182 (140)	972 (383)	790 (615 to 964)	0.005	168 (118)	180 (133)	12 (-65 to 89)	0.701
Total MET	566 (184)	1383 (402)	817 (630 to 1003)	0.005	530 (250)	520 (246)	−10 (-190.7 to170)	0.893
PCS (0-100)	49.3 (7.2)	52.8 (4.6)	3.5 (0.5 to 6.5)	0.018	50.5 (8.1)	50.9 (6.9)	0.38 (-4.22 to 4.99)	0.833
MCS (0-100)	50.3 (8.0)	53.1(5.4)	2.7 (-1.0 to 6.5)	0.074	51.0 (5.8)	51.8 (6.6)	0.8 (-3.2 to 5.0)	0.599
6MWT	555 (72)	584 (67)	29.4 (9.4 to 49.4)	0.001	554 (69)	562 (74)	8.2 (-16.7 to 33.2)	0.409

WMET = walking metabolic equivalent, total MET = total metabolic equivalent (extracted from International Physical Activity questionnaire), PCS = physical component score, MCS = mental component score, 6MWT = 6 minute walk test.

In addition, the total metabolic equivalent (T.MET) for time spent in vigorous, moderate and walking physical activity also showed a significant interaction, p < 0.005, F = 54.67 as well as group difference (p < 0.005; ES 2.59) by week 12 post-intervention. The intervention group significantly increased in T.MET (p < 0.005) with no significant changes in the control group (P = 0.889) at 12 weeks intervention (Table 2).

Analysis of the six minute walk test (6MWT) demonstrated a non-significant time by group interaction (p = 0.130; F = 2.05), with a significant increase between baseline and after the 12-week intervention (p < 0.005) in the intervention group compared to control group (p = 0.081) Table 2. Univariate analysis revealed that mean 6MWT was not significantly different between groups at 12 weeks intervention (p = 0.473; ES = 0.14).

### Health-related quality of life

There were no significant time by group interactions in MCS and PCS scores over time (p = 0.580; F = 0.536 and p = 0.072, F = 2.70 respectively). At 12 weeks, there were no significant differences between groups in MCS and PCS scores (p = 0.904; ES = 0.15 and p = 0.454; ES = 0.51 respectively). Despite the non-significant differences between groups in MCS and PCS scores, pairwise comparisons results indicate that the PCS scores significantly increased (p = 0.008), and there was a trend in significance in the MCS scores (p = 0.082) in the intervention group compared to the control group (p = 0.670 and p = 0.519 respectively) (Table 2).

### Anthropometric and physiological status

Between baseline and the completion of the 12-week intervention, significant improvements were observed in secondary health outcomes in the intervention group (Table 3). Small effect sizes were obtained for BMI (0.094), BF (0.081), DBP (0.032), SBP (0.030), and weight (0.093) and moderate effect size were obtained for WC and self-efficacy (0.272).

**Table 3:** **Secondary outcome measures: Mean (SD) of group changes, and mean differences within groups for outcomes between baseline and follow-up periods**

**12 week changes**			**Intervention n = 29**				**Control n = 29**	
	**Baseline**	**12 weeks**	**Mean difference (95% CI)**	**P value**	**Baseline**	**12 weeks**	**Mean difference (95% CI)**	**P value**
BMI	29.9 (7.2)	28.8 (6.8)	−1.1 (-2.3 to 0.1)	0.047	28.3 (4.4)	28.4 (4.3)	0.1 (-0.9 to 1.1)	0.856
S-E	2.6 (0.6)	3.1 (0.5)	0.5 (0.1 to 0.9)	0.007	2.7 (0.7)	2.8 (0.5)	0.1 (-0.2 to 0.4)	0.329
Body fat	29.6 (6.9)	27.9 (6.9)	−1.7 (-2.9to -0.4)	0.006	27.7 (6.9)	27.4 (6.5)	−0.4 (-1.2 to 0.6)	0.340
WC	98.9 (12.8)	97.1 (12.4)	−1.7 (-3.8 to 0.2)	0.029	93.5 (12.1)	93.8 (12.3)	0.2 (-1.3 to 1.8)	0.692
SBP	125.1 (16.4)	121.6 (12.7)	−3.5 (-7.4 to 0.4)	0.090	122.4 (11.4)	121.2 (9.4)	−1.1 (-4.7 to 2.4)	0.407
DBP	76.3 (9.7)	74.2 (7.3)	−2.1 (-4.7 to 0.6)	0.068	75.0 (6.7)	74.0 (5.2)	−1.0 (-3.2 to 1.2)	0.252
Weight	80.3 (16.9)	78.5 (16.2)	−1.7 (-3.9 to -0.4)	0.133	76.9 (16.2)	77.1 (12.8)	0.2 (-2.9 to 3.3)	0.886
**24 week changes**		**Intervention n = 29**				**Control n = 29**		
	**Baseline**	**24 weeks**	**Mean difference (95% CI)**	**P value**	**Baseline**	**24 weeks**	**Mean difference (95% CI)**	**P value**
BMI	29.9 (7.2)	29.0 (6.7)	−0.1 (-2.3 to 0.3)	0.062	28.4 (4.4)	28.5 (4.1)	0.1 (-1.2 to 1.4)	0.841
S-E	2.6 (0.6)	3.1 (0.5)	0.5 (0.2 to 0.8)	0.002	2.7 (0.7)	2.6 (0.5)	−0.1 (-0.4 to 0.3)	0.691
Body fat	29.6 (6.9)	27.9 (6.4)	−1.7 (-3.1 to -0.3)	0.011	27.8 (6.9)	27.6 (6.1)	−0.2 (-1.3 to 0.9)	0.661
WC	98.9 (12.8)	97.0 (12.3)	−1.9 (-3.9 to 0.1)	0.060	93.5 (12.1)	93.7 (12.2)	0.2 (-1.3 to 1.7)	0.741
SBP	125.1 (16.4)	120.9 (11.4)	−4.1 (-8.6 to 0.2)	0.030	122.4 (11.4)	121.5 (9.6)	−0.9 (-4.8 to 3.0)	0.572
DBP	76.3 (9.7)	74.1 (6.1)	−2.2 (-4.5 to 1.1)	0.165	75.1 (6.7)	74.4 (6.2)	−0.6 (-3.8 to 2.5)	0.608
Weight	80.3 (16.9)	78.7 (16.2)	−1.6 (-4.1 to 0.9)	0.330	76.9 (16.2)	77.5 (12.1)	0.6 (-2.6 to 3.8)	0.646

BMI = body mass index, WC = waist circumference, DBP = diastolic blood pressure, SBP = systolic blood pressure, S-E = self-efficacy.

## Discussion

The purpose of this study was to examine the feasibility and acceptance of using a pedometer-driven walking intervention programme, together with educational material and email support to increase ambulatory activity and improve health-related outcomes in a population of meat processing workers when compared to a control group from the same working population receiving educational material alone. This study has demonstrated that a workplace based pedometer-driven walking intervention programme was acceptable and achieved high adherence rates; it was also found to be feasible to recruit suitable adults from at least this workplace into a walking study. The intervention and methodology also proved successful based on the results of study adherence and the satisfaction survey, as well as the large effect size for the pedometer intervention on step-count level. In addition, no major issues arose within this setting with respect to wearing and use of pedometers to provide feedback towards step count goals.

Results of this feasibility study will be used to inform the development of a future fully-powered controlled trial to evaluate the effectiveness of this intervention in this population.

### Adherence with the intervention

Participants reported a high rate of adherence and retention to the walking programme over the initial 12-week period. Results show a higher rate of adherence compared with previous pedometer interventions reported in a variety of workplace populations, including university employees, [9,68,69] Home Depot workers [70], and office workers [71,72]. High adherence in the current study might be due to the high level of regular contact between participants with the principal researcher physically based on site, visiting participants in their own workspace environment in order to maintain follow-up contact in the study setting. Previous research has established that regular contact between participant and researcher [73] personal interactions [74] social support such as family or friend support, group programmes [75] and social communication in the workplace [10,76] can improve adherence to intervention programmes and motivate individuals to increase their physical activity levels.

The underlying reasons for the high level of adherence in our study are also likely to include the recruitment of participants who were inactive people at baseline. This is consistent with previous research conducted within workplace settings [10,77-79] that has identified that employees who start with low physical activity levels have a lower dropout level and a greater recorded increase in physical activity at intervention completion.

In the current study, participants reported that the pedometer was useful, very easy to use, and that it was enjoyable to see their progress of activity over the duration of the intervention. These positive results are consistent with similar walking studies conducted in a workplace setting [9,68,71,72,78,80].

### Intervention effect

The results of the current study revealed an increase in step count and quality of life scores in the intervention group; with slight increases in the control group also noted. The results correspond to approximately 30-40 minutes of increased walking per day. The increase in step count observed in the intervention group (3799 steps per day) was higher than has been typically reported in the literature for pedometer-based interventions in adults. A review of randomized controlled trials and observational studies of pedometer-based walking interventions (five studies were in workplace settings) in adult populations suggest that on average, pedometer-based interventions result in an increase of approximately 2000 to 2500 steps per day [24]. Previous research within workplace populations also reported moderate increase in steps ranging from 445 to 1120 steps per day [69,71,81-83]. However, the current intervention differs from these studies in several ways. These studies had recruited participants with higher levels of physical activity, and methodology and study designs also focused on both diet and physical activity. The mean daily increase in step count on completion of the intervention was 3799 steps per day (which is a mean 59% above baseline). This favourably compares to an average 26.9% noted in the systematic review of pedometer-based interventions in adult populations [24]. These authors suggest that pedometer-based interventions may be more effective at increasing physical activity when participants have a more sedentary lifestyle; those who are already physically active may find it more difficult to accumulate increased steps due to the time constraints of daily living. In the current study, using an individual baseline step goal to increase steps per day by 5% above previous values each week to reach a 10,000 steps per day target, appears to be feasible in increasing physical activity over the course of the walking programme. This target may have contributed to increased self-efficacy in the intervention group, helping them to increase their overall physical activity over the 12-week period. It has been proposed in earlier research that use of a common target of 10,000 steps per day can increase the level of physical activity [24,84]. Changes in the step counts and 6MWT were declined in the control group at three months post-intervention, the lack of improvement is not surprising. It might have decreased their physical activity due to the time constraints of daily living. This study also observed a significant intervention effect of weekly minutes of physical activity. In addition, large effect sizes were observed after the initial 12-week intervention period. These data suggest that a higher increase in step-count can confer a greater positive impact in increasing weekly minutes of physical activity as identified in the self-reported results for the International Physical Activity Questionnaire (IPAQ) which is consistent with previous research on physical activity [30,70,77] within the workplace. In addition, a ‘whole community’ intervention study [85] recruited 1242 participants across the East Flanders province of Belgium in 2005 with 68% of the sample being full-time employees. A significant increase in both pedometer and IPAQ self-reported physical activity were reported after one year with the majority of participants reporting a positive increase on IPAQ in the workplace setting.

Results from this study also showed significant improvements in physical component score (PCS) and non-significant improvements in mental component score (MCS) in the intervention group. These improvements are potentially due to the increase in physical activity in this group, and are consistent with findings from other studies [86-88] that reported improvements in HRQL after intervention periods. Positive associations between increased participation in physical activity and improved health-related quality of life are well published [87,89-92], highlighting that any increase in daily physical activity can confer wider health benefits. These findings are consistent with a recent systematic review of 13 studies including randomized controlled and controlled trials that investigated the effects of pole walking (PW) on HRQL and showed consistent positive associations between PW and HRQL in adults with and without clinical conditions [89].

In this study, significant improvement in several outcomes, including waist circumference, weight, body mass index, blood pressure, self-efficacy, and body fat was observed after completion of the 12-week intervention, with effect sizes ranging from small to medium. These findings are consistent with previous research that examined the impact of a pedometer-based walking intervention on health-related outcomes within workplace populations [30,71,77]. For example Chan et al. reported significant decreases in BMI, and waist girth (p < 0.001 for all) compared to the control [77]. Maruyama et al. [71] reported similar results after 12 weeks, whilst Morgan et al. [93] observed significant improvements in weight, waist circumference, BMI, and systolic blood pressure compared to the control group at the 14-week follow-up.

### Study limitations

As this is a feasibility study, there are limitations to the study that should be addressed in a full RCT in future. It was performed on a convenience sample of predominantly female subjects at a work site in a rural population. Therefore, these results may not be representative of an entire population and cannot be simply generalized to all meat workers in New Zealand. Future RCT studies are needed to compare different sites with larger sample sizes. In addition, participants were not blinded to allocation of the intervention, and also were able to monitor daily pedometer step counts throughout the seven day assessment periods. The non-concealment of the pedometer may have influenced step-count levels in both groups at assessment points; potentially this would have increased physical activity at these points and therefore reduced the effect between the groups.

### Study strengths

This study has several strengths; firstly, this is the first pedometer-based intervention, to our knowledge, conducted in New Zealand among meat processing workers. Therefore, the information and data on step count and other health parameters will provide direction for future pedometer intervention studies in a in a variety of settings. Secondly, we used a pedometer and IPAQ-short form to evaluate habitual physical activity at baseline and at follow-ups, which represent objective and subjective methods. We also chose an accurate pedometer available on the market based on clinical studies. This pedometer is a valid and reliable tool for counting steps in adults under free living condition [43,47].

## Conclusions

This study has demonstrated that a 12-week pedometer-based walking intervention in combination with goal setting, and self-monitoring supported by weekly e-mails, was feasible and potentially effective in increasing daily physical activity levels in low active meat workers. The pedometer-based intervention significantly increased physical activity levels and several outcomes, including physical component score, waist circumference, body mass index, and body fat compared to the control group. Walking is the most popular type of physical activity and is inexpensive and relatively simple to implement in the workplace, demonstrating a high level of adherence and good satisfaction to the intervention. The results indicate that increases in daily physical activity can confer improvements in the other health-related outcomes. More research, in a large randomized controlled trial study with long follow-up, is required to determine the true effectiveness of this intervention in a variety of settings.

## Abbreviations

RCT: Randomized controlled trial
MSDs: Musculoskeletal disorders
PA: Physical activity
PW: Pedometer-driven walking
CG: Control group
SFF: Silver Fern Farms
BP: Blood Pressure
BMI: Body Mass Index
BF: Body Fat Percentage
WC: Waist Circumference
SF-36v_2_: The Short Form 36 Version2 Questionnaire
IPAQ-SF: International Physical Activity Questionnaire
HRQL: Health Related Quality of Life
SW200: The Yamax Digi-walker pedometer SW-200(Yamax, Tokyo, Japan)
6MWT: The Six Minute Walk Test
PW: Pole Walking
NZ: New Zealand
SRT: Self-regulation theory
PCS: Physical component score
MCS: Mental component score
